# Supplementary material for: Are carbon emissions trading and green financial instruments synergistic? -Comprehensive quantitative research based on content analysis
Source: PLoS One. 2024 Mar 7;19(3):e0298601. doi: 10.1371/journal.pone.0298601 (PMC10919601; doi:10.1371/journal.pone.0298601)
Supplement: S1 Data — (ZIP) [file pone.0298601.s001.zip › Data/地级市Z_F工作报告.zip!/╕╩╦α/╢¿╬≈╩╨/└┤╫╘╣1⁄2╓┌║┼í╛┬φ┐╦╩2╛▌═°í┐_2015.docx]

各位代表:

　　现在，我代表市人民政府，向大会作工作报告，请予审议，并请各位政协委员和列席人员提出意见。

2014年工作回顾

　　过去的一年，面对严峻复杂的发展形势和持续加大的经济下行压力，在省委、省政府和市委的坚强领导下，全市上下认真贯彻落实党的十八大和十八届三中、四中全会精神及省、市一系列决策部署，牢牢把握稳中求进工作总基调，凝心聚力，攻坚克难，全面深化各项改革，经济社会发展呈现结构优化、质量提升的良好态势。全市完成生产总值267.94亿元，增长9.2%。固定资产投资500.59亿元，增长21.1%。规模以上工业增加值26.8亿元，增长10.2%。社会消费品零售总额92.4亿元，增长12.5%。大口径财政收入38.33亿元，公共财政预算收入21.54亿元，公共财政预算支出180.26亿元，分别增长25.05%、26.38%和1.9%。城镇居民人均可支配收入17216.9元，增长9.5%。农民人均纯收入4599.9元，增长12.6%。金融机构存贷款余额分别达到592.1亿元和428.9亿元，增长14.3%和36.1%，存贷比72.4%，较上年提高11.6个百分点。居民消费价格涨幅2.4%，人口自然增长率7‰以内，城镇登记失业率3.17%。

　　一年来，我们主要做了以下工作:

　　(一)奋力加快灾后重建，灾区面貌焕然一新。把岷县漳县地震灾后重建作为首要的政治任务和最大的民生工程，紧盯灾民建房和教育、卫生重建项目三大重点建设任务，定期调度，蹲点督查，跟踪落实，全力抓前期、保质量、赶进度，共组织实施重建项目1177项，完工397项，完成投资84.8亿元，占规划总投资的78.3%，年度建设任务全面完成，灾后重建取得阶段性重大成效。积极动员和组织干部群众采取结对帮建、互助共建、村组联建等多种方式共建美好家园，6.88万户城乡居民住房重建和7.96万户维修加固任务如期完成，靖远异地安置点居民住房主体工程基本建成，灾区群众居住条件发生历史性变化。建成教育项目158个、卫生项目60个，完工率分别达到87.3%和89.4%，83所帐篷学校全部拆除，灾区学校秋季如期开学。岷县梅川茶埠新区、漳县大草滩等7个小城镇、48个集中安置点及公共服务项目有序推进，水利、交通等基础设施项目启动实施。及时筹措下拨冬春救助资金，妥善安排困难群众生活，灾区学校按期供暖，居住在帐篷里的受灾群众全部搬入新居，实现温暖安全过冬。结合美丽乡村建设，对梅川、茶埠、三岔、五竹等公路沿线重点小城镇实施风貌改造，对44个乡镇、98个村庄开展环境综合整治，灾区风貌大为改观，面貌焕然一新。

　　(二)全力加强项目建设，拉动经济平稳增长。深入实施“3341”项目工程，紧盯国家政策投资导向，谋划生成项目5218个，总投资8276亿元。争取国家和省上各类项目资金75亿元。我市被确定为第一批国家生态文明先行示范区，引洮二期工程项目建议书获国家批复。实施500万元以上项目1498个，同比增加130个，其中亿元以上278个，增加87个。宝兰客专、兰渝铁路等16个省列重大项目加快推进，西部国际汽车商贸城、奇正藏药无公害特色中药材深加工等150个市列重点建设项目完成年度投资计划，列入重点前期项目的华盛影视文化旅游创意园开工建设。引洮供水一期工程主干渠全线贯通，定西市支渠、城镇供水及部分农村供水工程同步试通水，已有66.27万城乡群众喝上了甘甜纯净的洮河水，全市人民期盼半个多世纪的引洮梦终于实现。扎实推进“千亿元大招商”活动，重点围绕生物医药、装备制造、化工建材、现代物流等主导产业，瞄准国内知名企业开展一对一、点对点精准招商，长江三峡新能源、天津亿联集团等大型企业已到定西投资兴业。加大节会推介力度，加强考核管理，强化跟踪服务，狠抓落地建设，第20届“兰洽会”签约项目152个，总投资614.93亿元，到位资金180.36亿元，到位率29.3%。全市实施招商引资项目420个，到位资金417亿元，增长40.1%。认真落实注册资本登记制度改革，扶持非公有制经济加快发展，新增非公企业2512户、个体工商户1.23万户，全市各类市场主体达到9.87万户，非公经济完成增加值126.37亿元，占生产总值的46.23%。

　　(三)坚持综合精准施策，致力推进扶贫攻坚。扎实推进“1236”扶贫攻坚行动，落实各项精准扶贫措施，全市有10个重点乡、139个重点村整体脱贫，减少贫困人口16.5万人，贫困面下降到26%。争取财政专项扶贫资金3.65亿元，较上年增长33.6%。制定出台配套政策22项，全面完成869个贫困村、83.92万贫困人口调查摸底和建档立卡工作。深化“双联”行动，建立“一村一业一单位、一户一策一干部”结对帮扶机制，共有1081个单位、4.15万名干部结对帮扶19.02万贫困户，实现了所有贫困户帮扶干部全覆盖。持续改善贫困村基本生产条件，通渭和临洮交通扶贫攻坚试点县建设快速推进，全市建成农村公路1019.5公里，行政村通畅率达到67%。实施重点水利工程19项，解决了10万人的饮水安全问题，农村自来水入户率达到70%。农村电网综合改造率和自然村动力电覆盖率分别达到86%和89%。易地扶贫搬迁贫困群众1.3万户6.8万人，改造农村危房2.39万户，新建农村户用沼气1740户。互助增信平台试点初见成效，新增贫困村互助资金协会156个、累计达到480个，资金总量达到9708.5万元，贫困群众贷款难问题得到有效缓解。完成贫困家庭“两后生”培训9000人，培训农村劳动力22.3万人，输转劳务68.15万人次，其中组织输转42万人次，创劳务收入98.5亿元。

　　(四)着力调整优化结构，推动经济转型升级。立足优势补短板，全力优化产业结构，三次产业结构调整为29:25:46，一产较上年下降2个百分点、二产下降0.6个百分点、三产上升2.6个百分点，经济转型升级步伐加快，发展质量有效提升，带动地方财政收入加快增长，公共财政预算收入增速高于生产总值增速近18个百分点。

　　围绕发展现代农业，全力推动中医药、马铃薯产业转型升级，草食畜牧业和蔬菜产业发展壮大。全市种植中药材136万亩，总产量、饮片加工能力均达到30万吨，种植加工总产值113亿元。中药材静态仓储能力80万吨，年交易量90多万吨，完成交易额249.5亿元。康美西部中药城开工建设，陇西“全国中药材种植产业知名品牌创建示范区”获批筹建，甘肃陇西中医药产业研究院挂牌成立。种植马铃薯288.6万亩，总产量、贮藏能力、加工能力分别达到500万吨、350万吨和60万吨，实现总产值120亿元。组建马铃薯种薯行业协会，建成省级以上种薯综合标准化示范区3个，生产原原种7亿粒。安定“全国马铃薯产业知名品牌创建示范区”通过国家专家组现场验收。中药材和马铃薯及其制品2个省级质检中心基本建成。大力发展草食畜牧业，畜禽饲养总量达到2440万头(只)，增长19.4%，肉蛋奶产量达到27万吨，增长17.4%，实现总产值120亿元。财政投入资金1.16亿元，扶持发展养羊示范户2.05万户，新建规模养殖场(小区)480个、家庭养殖场2470个，引进500万元以上畜牧企业37家，完成玉米秸秆青贮氨化260万吨。新发展设施农业2万亩，累计达到12.15万亩，蔬菜种植面积达到54.7万亩，实现总产值61.5亿元。推广旱作农业394万亩，发展高效节水农业61万亩，粮食生产实现“七连丰”，总产量达到158.6万吨。

　　深入实施工业发展倍增计划，努力扭转工业经济下滑态势。实施千万元以上工业项目215个，其中甘肃招金难处理金精矿冶炼等45个建成投产，天信印务包装纸箱生产等170个完成年度建设任务，完成工业固定资产投资120亿元，增长15%。加快推进经济开发区和工业集中区循环化改造，积极承接产业转移，新入驻企业68户，园区完成投资138亿元，实现工业增加值28.5亿元，占全市工业增加值的67.8%。积极开展技术创新，不断改造提升传统产业，实施甘肃宏腾特种车辆加工改扩建等高新技术改造项目186项，组建市级工程技术中心111家，3家企业技术中心通过省级认证，全市规模以上工业企业基本实现工程技术中心全覆盖。加大企业培育扶持力度，新增规模以上工业企业25户、产值过亿元企业8户。市财政安排资金1000万元建立“助保贷”融资平台，全力助推小微企业发展。巨鹏食品、凯凯薯业、清吉淀粉、中天羊业等77户企业分别在省内外实现股权挂牌交易。

　　抢抓“丝绸之路经济带”甘肃黄金段和华夏文明传承创新区建设机遇，全力发展文化旅游产业。渭河源、李家龙宫景区分别被纳入全省丝绸之路旅游经济带大景区、重点景区建设规划，贵清山遮阳山游客接待中心全面建成，临洮马家窑文化产业园等35个重点项目加快推进，累计完成投资35.66亿元。定西剪纸等3个项目入选国家级非遗项目名录，岷县《巴当舞》获全国民间文艺“山花奖”金奖。成功举办中国·陇西第三届世界李氏文化旅游节暨2014年全球李氏恳亲大会、马家窑文化国际学术论坛暨马家窑遗址发现九十周年纪念大会、2014甘肃·渭源大禹公祭大典等大型节会。全市文化产业增加值4.98亿元，增长32%；旅游综合收入15.5亿元，增长25%。大力发展现代物流业，陇西桥南家居建材城等项目建成运营，荣芳汽配城、西北金泽物流园等项目进展顺利，陇西首阳产地药材交易市场等3个大型农产品批发市场完成年度建设任务。新型业态快速兴起，建成陇西“药财盈”等电子商务交易平台10个，全市网店发展到800多家，50家企业产品入驻“淘宝网”甘肃馆，实现零售额4000多万元。民营快递业迅速发展，110多家企业及其分支机构落户我市。甘肃银行陇西、临洮、渭源、岷县支行顺利开业，兰州银行设立安定、岷县支行，临洮、定西农村商业银行组建运营，邮储银行通渭支行完成改革，金融便农服务网点发展到2384个，基本实现城乡基础金融服务全覆盖。

　　(五)加快新型城镇化建设，统筹城乡一体发展。积极推进以人为核心的新型城镇化建设，坚持以城带乡，加快城乡一体化发展步伐。有序推进农业转移人口市民化，加快户籍制度改革，全市城镇化率达到29.23%，较上年提高2.07个百分点。陇西县被列为全省新型城镇化建设试点县，安定区巉口镇、通渭县马营镇、漳县大草滩(殪虎桥)乡、岷县梅川(茶埠)镇被列为试点镇。加强城乡规划管理，定西市城市总体规划启动修编，村庄规划编制覆盖率提高到85%。实施各类城建项目257个，完成投资109.7亿元。市区实施项目72个，完成投资48.9亿元，城市功能日趋完善，城市品位进一步提升。新城区电网工程等项目建成投入使用，市文化艺术中心、体育馆、游泳馆等项目加快推进，市行政中心、市建设工程服务中心等单位实现整体搬迁，新城区聚集效应开始显现。旧城区西岩新村片区改造、南环路改造等项目完成年度投资计划，市区交通路改造工程建成投入使用，有效缓解了通行压力。各县城实施项目185个，完成投资60.8亿元，综合承载和辐射带动功能不断增强。修订完善棚户区改造规划，争取国开行贷款授信额度30.65亿元。重视解决群众反映强烈的物业管理、供热服务、环境卫生和交通秩序等问题，市区新建过街天桥2座、公交站点65处，新开通连接新旧城区公交线路3条，新投放更换公交车75辆，方便了群众出行。加强生态文明建设，全力改善农村生产生活条件，实施农村环境连片整治项目47个，申报创建国家级生态乡镇2个、省级7个。新修梯田28.5万亩，新增水土流失治理面积350平方公里。实施重点林业工程26.6万亩，建设优质林果基地9万亩。实施土地开发整理项目21.57万亩，新增耕地2.87万亩。

　　(六)全面深化各项改革，不断增强发展活力。顺利完成市、县区政府机构改革，共精简机构22个，精简率11%。推行政府及部门权力清单制度，共承接省级下放行政审批事项50项，市级分4批调整取消下放行政审批事项90项。新的市政府政务服务中心建成运行，首批入驻单位47个，开展审批服务事项388项。强化行政审批项目监管，编制了《定西市现行保留行政审批项目目录》，制定了《定西市行政审批事项目录管理办法》。全力推进公共资源交易市场化配置改革，规范各类公共资源交易活动。推行阳光政务，市县区财政部门及所有预算单位预决算和“三公”经费信息全部公开。深化医药卫生体制改革，完成市级卫生计生机构整合，市医院加入兰大二院医疗集团合作办院并迁入新址正式运营，通渭、陇西、临洮三县公立医院改革试点进展良好。完善现代农业经营体制，流转土地38万亩、林地1.75万亩，新增家庭农场73个，陇西云田、临洮新添省级农村土地承包经营权确权登记颁证整乡试点工作进展顺利。中药材产值保险试点承保面积8.77万亩，药农获赔3500万元，试点工作被中国改革年会评为2014年度中国改革10大案例之一。陇西渭水酒业集团完成改制，新组建的陇西天马酒业公司投产运营。

　　(七)大力保障改善民生，不断增进群众福祉。财政用于民生方面支出97亿元，增长12.5%。省、市为民办理的实事全面完成。千方百计扩大城乡就业，城镇新增就业3.08万人。新增小额担保贷款3.21亿元，扶持8300多人自主创业，带动就业1.68万人。狠抓教育教学质量提升，高考二本录取率29.5%，应届生上线率提高3.57个百分点。着力改善办学条件，改造新建校舍34.9万平方米，消除D级危房28万平方米，乡镇公办幼儿园实现全覆盖。加强教师队伍建设，强化业务培训，引进重点师范院校毕业生47名，为20119名乡村教师发放了生活补助。积极申报筹建定西职业技术学院，定西师专建设甘肃中医学院定西校区工作进入移交阶段。新农合参合率达到97.08%，在全省率先试点的部分住院病种分级诊疗改革顺利推进，病人转外就医率为13.5%，居全省最低。大病保险试点成效显著，为2.07万患者报销医药费8295万元，大病患者实际报销比例由48.12%提高到62.88%。有序实施“单独两孩”政策，低生育水平持续稳定。城乡居民社会养老保险实际参保153.67万人，参保率达到95.4%。在全省率先建立被征地农民养老保险准备金制度。投入资金2.13亿元，帮扶6.3万户计生贫困家庭发展。全力保障困难群众基本生活，城乡低保标准分别提高15%，发放临时救助和城乡医疗救助1.15亿元。积极推动军民融合深度发展，国防动员和双拥工作得到加强。实施科技惠民示范工程7项，强化科技成果转化应用，中药材气调保质贮存专利技术示范应用效果明显，填补了国内空白。大力实施明厨亮灶工程、文明餐桌行动和食品安全状况“外置化”管理，深入开展农村食品市场、药械质量安全专项整治，严厉打击食品药品违法犯罪行为，全市未发生药品安全事故和较大以上食品安全事故。不断强化安全生产“红线”意识，认真落实“党政同责、一岗双责”，安全生产形势总体比较平稳。大力开展非正常上访和命案防控专项整治，深入推进涉法涉诉信访制度改革，超前化解防范各类矛盾纠纷和信访难题，全面构筑立体化防控体系，“两个全员参与”机制得到省委、省政府充分肯定，农村基层规范治理有了新提升，社会大局保持稳定。民族宗教、外事侨务、审计、地震、粮食、气象、通信等工作，以及妇女、儿童、老年人、残疾人、社会慈善等事业都取得了新的成绩。

　　(八)切实加强自身建设，努力提高行政效能。坚持落实市政府常务会议会前学法制度和规范性文件合法性审查报送备案制度，不断提高决策的科学性和合法性。严格执法责任，强化执法监督，规范执法行为，积极探索综合行政执法改革，依法行政效能进一步提高。深入开展党的群众路线教育实践活动，新制定和修订完善市政府工作规则、重大行政决策程序规定、专项资金管理办法等制度22项，进一步规范了行政行为。下决心整治灾后重建、城乡低保、危房改造、扶贫资金等重点领域存在的突出问题，坚决纠正执法部门、窗口单位门难进、脸难看、事难办和慵懒散慢拖等不良作风，处理问责干部140人，政风行风明显好转。认真执行中央八项规定、国务院“约法三章”和省、市相关规定，严格落实党风廉政建设主体责任，着力解决“四风”突出问题，会议、文件减少20%和15%，“三公”经费支出下降29.3%，年内没有新建楼堂馆所。按照市委统一部署，紧紧围绕精准扶贫、灾后重建、产业发展等事关全市发展的重大问题，深入开展大调研活动。定期向市人大及其常委会报告工作，主动加强与市政协联系，108件市人大代表建议和192件市政协委员提案按期办结。

　　各位代表！回顾过去一年，遇到的困难前所未有，发展的任务异常繁重，取得的成绩令人鼓舞。这是市委坚强领导的结果，是市人大、市政协监督支持和全市人民奋斗拼搏的结果。在此，我代表市人民政府，向奋战在各条战线的广大干部群众，向各人民团体、民主党派、工商联和无党派人士，向人民解放军、武警官兵和中央、省驻定单位，向所有关心支持定西发展进步的各界人士，表示衷心的感谢和崇高的敬意！

　　各位代表！当前定西正处在全力扶贫攻坚、经济转型升级的关键时期，我们清醒地认识到，全市经济社会发展仍然面临一些突出困难和问题。主要是:农业和农村发展条件依然薄弱，贫困区域基础条件差，群众增收渠道单一，持续增收难度较大；市场有效需求不足，企业效益下滑，循环经济产业链条不长，经济下行压力加大；优势特色产业发展面临增长与结构调整、壮大与转型升级双重压力；财政收支矛盾突出，市场主体融资比较困难，用地、资金等要素制约日益凸显；科技研发和自主创新能力不强，推动科技创新、培育新的经济增长点还需下更大功夫；社会治理难度加大，保障和改善民生的任务还十分繁重；政府职能转变还不到位，个别工作人员责任意识不强、服务意识不足、工作效率不高、抓落实不力等问题依然存在。对此，我们将采取切实有效措施，认真加以解决。

2015年主要任务

　　2015年，是全力深化改革的关键之年，是全面推进依法治市的开局之年，也是实施“十二五”规划的收官之年。当前，我国经济增长进入换挡期、结构调整面临阵痛期、前期刺激政策处于消化期，经济发展的速度、方式、结构、动力都发生了新的变化。从我市来看，也呈现出一系列新的特征，主要体现在经济由高速增长向中高速增长转变，扶贫开发模式由传统单一向综合创新转变，优势特色产业发展由速度规模型向质量效益型转变，产业结构由传统产业为主向传统产业转型升级与战略性新兴产业快速兴起转变，经济增长动力由投资拉动为主向投资消费并重转变，招商引资由主要依靠优惠政策向注重营造良好环境转变。对市情的深刻认识是做好今年经济工作的前提，我们必须适应好、把握好、引领好经济发展新常态，自觉做到因势利导、趋利避害，牢牢把握经济工作主动权。

　　2015年政府工作的总体要求是:全面贯彻落实党的十八大及十八届三中、四中全会精神，省委、市委全委(扩大)会议暨经济工作会议精神，深入学习贯彻习近平总书记系列重要讲话，主动适应经济发展新常态，坚持稳增长、调结构、转方式、惠民生，大力发扬“三苦”精神，着重在扶贫攻坚、项目建设、创新驱动、产业升级、深化改革、民生保障方面实现新突破，促进全市经济平稳健康发展、社会和谐稳定。

　　经济社会发展的主要预期目标是:生产总值增长8%，固定资产投资增长20%，规模以上工业增加值增长10%，社会消费品零售总额增长12%，大口径财政收入、公共财政预算收入均增长12%，城镇居民人均可支配收入增长10%，农民人均纯收入增长13%。居民消费价格涨幅控制在3%以内，人口自然增长率控制在7.1‰以下，城镇登记失业率控制在4%以内，节能减排完成省下达目标。

　　以上预期目标，主要是考虑了两个方面的因素。一是考虑了改革发展大势和国家政策导向。我国经济发展已经进入中高速增长的新常态，今年国内经济下行压力会继续增大，国家和省上适度下调预期目标，我们也相应作了下调安排，使经济运行保持在合理区间。二是考虑了我市的阶段性发展特征。我市正处于奋力扶贫攻坚、加快经济转型升级的关键时期，需要自我加压、奋力赶超、缩小差距。

　　围绕上述要求和目标，今年重点抓好以下工作:

　　(一)聚力抓好扶贫攻坚。深入实施“1236”扶贫攻坚行动，按照“五个精准”和“四个到村到户”要求，创新完善精准扶贫机制，年内148个贫困村实现整体脱贫，减少贫困人口12.5万人。把改善基础条件作为扶贫攻坚的当务之急，积极整合各类项目资金，谋划实施一批水、电、路、房等项目。全面完成通渭、临洮县交通扶贫攻坚试点任务，率先实现行政村100%通水泥(沥青)路目标。实施重点水利工程32项，农村安全饮水入户率达到80%以上。实施农村电网改造工程，贫困村动力电覆盖率达到95%。争取实施整村推进项目75个以上，易地扶贫搬迁贫困群众6000户、2.8万人。抓好中国扶贫纪念馆建设，力争早日投入使用。把培育富民产业作为扶贫攻坚的核心任务，转变农业发展方式，调整种养结构和产品结构，因地制宜发展中医药、马铃薯、蔬菜等优势特色产业，花卉、林果、小杂粮等富民多元产业，动员千家万户大力发展以养羊为主的草食畜牧业，促进贫困群众人均纯收入增长15%以上。把提升贫困群众能力素质作为扶贫攻坚的治本之举，研究制定贫困家庭学生教育扶助政策，大力发展职业教育，培训“两后生”9000人，输转劳动力63.1万人次，实现劳务收入100亿元以上。把强化金融支持作为扶贫攻坚的重要保障，全力落实中和农信小额信贷、双联惠农贷款、世行第六期贷款和国家贴息扶贫贷款政策，探索多渠道融资办法，发挥互助增信平台作用，解决贫困群众发展资金短缺问题。把落实低保政策作为扶贫攻坚的有效补充，推进农村低保与扶贫开发政策紧密衔接，全市一类、二类低保对象和五保户保障标准提高到贫困标准线以上，实现一次性政策脱贫。把社会帮扶作为扶贫攻坚的助推手段，深化“双联”行动，动员社会各方力量参与扶贫开发，积极与国家部委、大型企业、发达地区加强对接，着力构建一体化扶贫大格局。

　　(二)全力加快灾后重建。举全市之力打好灾后重建攻坚战，把交通、水利项目作为重中之重，倒排工期，强化措施，挂图作战，确保按规划完成年度建设任务。加快项目前期规划设计进度，确保3月底前完成所有已下达投资计划项目的各项准备工作，4月份全部开(复)工建设。全力抓好城乡居民住房厨厕墙、水电路等配套设施和教育、卫生项目扫尾工程建设。5月底前完成靖远异地安置点住房、公共服务配套设施建设任务，6月底前搬迁入住。7月底前完成住房验收、集中安置点配套设施建设和教育、卫生完工项目审计验收。8月底前完成重灾村配套基础设施和未完工中小学、幼儿园等教育项目建设任务，秋季开学投入使用。10月底前完成人饮工程、堤防等水利项目、地质灾害治理项目和农村公路重建任务。11月底前完成小城镇配套基础设施建设任务。年底前全面完成文化广电、社会福利等公共服务设施建设，基本完成国省干线公路、产业扶贫、生态治理等重建任务。加强重建项目资金和质量监管，严格执行资金申报、审核拨付、公示公开、监督检查等制度，力争年底前基本完成竣工项目审计验收工作。

　　(三)大力深化各项改革。坚持问题导向，紧紧围绕制约经济社会发展最突出的问题和最薄弱的环节，鼓励基层主动探索、大胆创新、破壁拆墙，以问题的有效解决倒逼改革加快推进。积极开展综合行政执法改革试点，按照减少层次、整合队伍、提高效率的要求，构建层级之间合理分工、协调运作的工作机制，推动执法重心向基层下移。深化财税金融体制改革，完善政府预算体系，改进预算控制方式，严格政府债务管理，清理规范各类财税优惠政策，完善行政事业性收费和政府性基金管理政策。加快公共资源交易信息化平台建设，进一步规范交易行为。落实“营改增”试点政策，统筹做好资源税、消费税等税制改革工作。继续推进农村金融服务综合改革试点，加快农村信用社改制，大力发展村镇银行。鼓励支持银行业金融机构向基层布点，加大边远山区金融便民点建设力度。创新各类产业扶持、支农和扶贫资金使用方式，更多用于担保贴息、风险补偿和企业股权投资，撬动更多金融和社会资金投入“三农”和产业发展。积极推广草食畜牧业保险贷款、中药材仓单质押贷款等金融产品，开展农村土地承包经营权抵押贷款和互助增信平台反抵押贷款试点，扩大农户小额信贷覆盖面，解决“三农”融资难题。进一步完善中药材产值保险工作，逐步在全市范围内推广。探索开展农房、牛羊养殖、蔬菜种植保险等涉农保险。加强对投资公司、担保公司等机构的日常监管，依法严厉打击非法集资等金融犯罪行为，营造良好的金融生态环境。大力推进县级公立医院综合改革，创新医保支付、多点执业、分级诊疗、双向转诊等机制，推进基本公共卫生服务均等化和市县乡村医疗服务一体化。加快推进农村综合改革，抓好陇西农村集体产权股份合作制改革试点，全面开展农村土地承包经营权确权登记颁证试点工作，鼓励农民以土地经营权入股合作社和龙头企业，发展多种形式适度规模经营。积极推进集体林权制度综合配套改革和林下经济发展。

　　(四)强力推进项目建设。紧盯“丝绸之路经济带”甘肃黄金段、华夏文明传承创新试验区、国家生态文明先行示范区建设等重大机遇，编制和谋划储备一批打基础、管长远的重大规划和重大项目。年内争取国家和省上各类项目资金50亿元以上。实施500万元以上项目1600个，力争完成投资600亿元以上。争取陇西一方制药等重点企业纳入省级战略性新兴产业支持项目库，积极助推临洮机场军民合用改扩建、定西热电联产等项目前期工作取得新进展。配合抓好兰渝铁路、宝兰客专、临渭高速、渭武高速、兰州至定西天然气管道等省部属重大项目建设。全力组织实施市列领导责任制重点项目。积极推进“多规合一”规划体制改革，全面完成“十三五”规划纲要及30项市级重点专项规划编制工作。深入推进“千亿元大招商”活动，坚持引资、引技、引智有机结合，主动与大企业、大集团对接，强化招商引资目标管理责任，年内项目开工率达到85%以上，资金到位率20%以上，到位资金比上年增长25%，达到520亿元。主动研究分析招商引资工作面临的新趋势，在全面落实优惠政策、完善承载功能的基础上，把工作重心转到优化服务和营造环境上来，从关联产业配套、人力资源开发、企业投资融资、居家环境改善、上学就医消费等方面创造条件、提供服务，努力营造良好的投资环境，提高招商引资的吸引力和竞争力。

　　(五)着力做大优势特色产业。认真实施全省“365”现代农业发展行动计划，围绕引洮一期水资源综合高效利用，全力推动中医药、马铃薯产业转型升级和草食畜牧业、蔬菜产业发展壮大，着力调整优化农业结构。加快推进中医药产业标准化建设，建立标准化药源基地70万亩、种子种苗繁育基地6万亩。进一步规范中药材市场交易秩序，积极筹建定西中药材商品交易所，推动市场交易向现代化电子交易转变。大力推广中药材气调保质贮存技术和作业标准，不断提高中药材仓储质量和水平。争取党参、黄芪列入药食同源目录，加快发展中药健康产业。年内建成陇西“全国中药材种植产业知名品牌创建示范区”，并通过国家质检总局验收。中医药产业种植及加工总产值达到130亿元。稳定马铃薯种植面积，强化种薯质量及市场监管，大力推进标准化生产，推动种薯产业转型升级。建立标准化种植基地270万亩，原种扩繁基地6万亩，一级种、二级种扩繁基地150万亩，生产脱毒原原种8亿粒。抢抓马铃薯主粮化战略机遇，尽快研发生产适应市场需求的多元化产品。充分发挥甘肃定西马铃薯循环经济产业园聚集功能，吸引国内外知名食品加工企业入园发展。依托甘肃省马铃薯工程技术研究中心等科研机构，加快马铃薯清洁生产技术开发及推广应用。健全完善马铃薯市场体系，加快定西国家级马铃薯批发市场建设，扩大安定“全国马铃薯产业知名品牌创建示范区”带动效应。加快建设陇药文化馆和马铃薯博物馆，提升规划设计标准，加快布展进度，确保及早投入运行。以肉羊产业发展为主导，扶持养羊示范户2万户以上，新增家庭养殖场1000个、规模养殖场(小区)300个、养殖专业村100个。引进500万元以上畜牧企业20家，各县区至少建成1个综合畜禽交易市场，完成玉米秸秆青贮氨化280万吨，力争畜牧业增加值占农业增加值的比重达到30%。加快发展蔬菜产业，以全市57个蔬菜产业重点乡镇为主，统一规划、合理布局，新增设施农业2万亩，蔬菜种植面积达到70万亩。大力推广标准化农膜生产应用，完成全膜双垄沟播种植371万亩，粮食总产量稳定在150万吨左右。

　　(六)倾力发展新型工业。围绕打造千亿级产业、百亿级园区、十亿级企业，深入推进工业发展倍增计划。加快主导产业结构调整，积极争取实施高新技术改造项目，推动有色冶金、矿产建材、装备制造、马铃薯精深加工等传统产业改造升级，促进全产业链集群发展。重点抓好通渭华家岭20万千瓦风电、中庆建材公司低辐射镀膜玻璃生产线等千万元以上工业项目138个，力争天信印务包装纸箱、甘肃润华压力容器生产基地等48个项目建成投产，工业固定资产投资增长15%。加快国家中医药产业发展综合试验区试点市建设，扶持岷海、扶正等企业先行先试，率先实现中药配方颗粒批量生产。新建中药提取物生产线8条，中药材饮片加工能力扩大到35万吨。积极研发膳食饮片、药妆等产品，提高产品附加值和市场竞争力。进一步优化定西经济开发区功能定位，理顺管理体制，提升开发区建设管理水平，促进产业集聚发展。完成定西、陇西、临洮、岷县4个省级开发区循环化改造任务。积极承接产业转移，加强企业入园和项目落地，完成投资160亿元以上，增长18%以上，新入驻企业90户。对经济开发区和工业集中区内长期占地不开工的企业进行清理整顿，依法收回土地，进行二次招商，提高土地集约利用水平。认真落实扶持中小微企业发展优惠政策，鼓励有实力的企业上市融资，逐步建立以甘肃生物医药产业创投基金为主，其他基金、股权交易市场等共同参与，项目、信贷、保险、税收等全方位扶持的中小企业培育机制。充分发挥中小微企业融资“助保贷”平台作用，集中力量培育发展前景较好企业快速成长，新增产值过亿元企业3户、规模以上工业企业20户。健全完善融资等公共服务体系，加强运行监测，帮助停产半停产企业尽快恢复生产。鼓励企业开展科技创新，推动企业创新能力和品牌建设，争创省级及以上技术中心3家、省级和国家级技术创新示范企业2户、中国驰名商标1件、省著名商标8件、甘肃名牌产品7个。以推进“三转”为重点，认真落实扶持非公经济发展的各项政策措施，进一步降低行业准入门槛，优化发展环境，非公经济增加值增长16%，新增各类市场主体1.4万个、新增从业人员4万名。

　　(七)致力做活第三产业。牢牢把握扩大内需战略基点，坚持生产性服务业和生活性服务业双轮驱动、传统服务业和现代服务业齐头并进，努力促进第三产业发展提速、比重提高、水平提升。重视文化传承与文化遗产保护，重点抓好中国彩陶博物馆、马家窑博物馆等“四馆”建设，开展市县文化资源普查和分级分类评估工作。完善公共文化服务体系，重点抓好市图书馆、博物馆、城市展览馆、引洮工程纪念馆等“八馆”建设，建成“乡村舞台”343个。围绕华夏文明传承创新区建设，大力发展文化旅游产业，抓好临洮马家窑、通渭悦心、岷县洮砚等文化产业园和渭河源、李家龙宫大景区及贵清山植物园等重点项目建设，新发展农家乐100户以上，文化产业增加值增长32%，旅游综合收入增长25%。编制完成全市商贸物流产业发展规划，科学合理建设县域物流中心和区域物资集散地。加快重点商贸物流项目建设，启动实施安定南川工业园“退二进三”工作，力争定西亿联国际商贸城、九洲通现代中医药物流园尽早开工建设，马铃薯交易中心、陇西文峰中药材交易城和首阳产地药材交易市场等项目完成年度建设任务，荣芳汽配城、西部国际汽车商贸城销售功能区建成运营。推进电子商务由农特产品网上交易向各个领域拓展，促进快递物流与制造业及关联产业联动发展，带动社会消费增长。围绕实施养老健康家政、信息、旅游、住房、绿色、教育文化六大领域消费工程，打造多点支撑的消费格局，服务业增加值增长8.5%。

　　(八)合力统筹城乡建设。以全省新型城镇化试点县、试点镇建设为契机，着力完善市区、县城和乡镇三级城镇体系，不断增强综合承载功能，积极引导农业转移人口市民化，城镇化率达到30%以上。完成定西市城市总体规划修编，力争实现市区、县城、国家级和省级重点小城镇控制性详细规划全覆盖。围绕建设新型中等化城市，合理布局市区行政办公、商业服务、文化娱乐、教育卫生、住宅居家等功能分区，着重促进新城区聚集人气、旧城区拓展空间、开发区集聚产业，形成新旧城区和经济开发区融合一体发展新格局。年内实施城建项目90个，力争完成投资50亿元。新城区重点实施亚行贷款城市道路、景观绿化等基础设施项目，全面完成体育馆建设工程和市展览馆、市文化艺术中心内外装饰工程，确保中和教育世家、海旺家园等住宅小区建成交付使用，福台中学和西北师大附属中和学校如期招生，规划建设古玩、美食等专业市场和儿童游乐、健康养老等设施。旧城区重点抓好交通路南段拓宽改造、供热管网改造、背街小巷环境治理等基础设施项目，加快实施东关市场、永定市场和铭峰华府等旧城改造项目，打通连接旧城区、新城区和开发区之间的断头路，实现道路交通顺畅连接。强化电力通讯、供水排水、污水收集等设施建设，完善城市功能。不断强化城市管理，改进物业和供热服务，推进城市管理向街道和社区延伸，构建网格化管理格局。大力整治交通秩序和环境卫生，解决占道经营、以路为市、流动摊贩等突出问题，营造整洁优美的市容环境。扎实开展改善农村人居环境行动，高标准打造10个以上“千村美丽”示范村，完成63个乡镇和145个村庄环境综合整治任务。加强生态文明建设，积极争取项目资金，启动实施《渭河源区生态保护与综合治理规划》。落实最严格水资源管理制度，关闭引洮供水一期工程受益区取水井，加强河道监管。健全完善引洮一期配套工程运行管理机制，确保田间配套工程年后投入春灌，6月底前完成农村供水工程试通水工作。加快引洮供水二期工程前期工作，配合省上争取及早开工建设。加大封山禁牧力度，大力实施退耕还林等重点生态工程，巩固提升平定、天定等高速公路生态长廊建设成果，开展全民义务植树，整区域推进造林绿化，完成造林封育24万亩，其中退耕还林12万亩、市区面山绿化4万亩，森林覆盖率达到12.6%。新修梯田30万亩，治理水土流失面积350平方公里。开展“三沼”综合利用技术推广，加强农业面源污染治理，废旧农膜回收利用率和尾菜处理利用率分别达到78%和30%。

　　(九)努力保障和改善民生。认真落实促进就业的各项政策措施，鼓励支持大众创业，城镇新增就业2.58万人。积极推进城乡居民基本养老保险和被征地农民养老保险工作。大力发展现代职业教育，争取启动建设定西职业技术学院，每个县区创建一所特色职业技术学校。加强学校内部管理，加大教师培训力度，坚持不懈提升教育质量，高考应届生二本上线率提高3个百分点。以义务教育标准化学校建设为抓手，努力改善办学条件，促进义务教育全面均衡发展。注重发展学前教育，积极实施特殊教育提升计划。实施县乡村卫生计生机构标准化建设工程，加快创建中医药工作先进市县。完善计划生育利益导向和优质服务机制，提高人口素质，稳定低生育水平。广泛开展全民健身运动，促进群众体育和竞技体育全面发展。逐步扩大住房公积金政策覆盖面，严格按要求提高缴存比例。认真落实安全生产监管责任和企业主体责任，夯实工作基础，创新监管机制，促进安全生产形势稳定好转。强化市场监管，规范市场经济秩序。推进食品药品生产经营使用规范化建设，进一步落实各级监管责任，保障人民群众饮食用药安全。深入开展公民道德教育，加强和创新社会治理，统筹基层政法资源，巩固完善“两个全员参与”机制，合力化解矛盾纠纷和信访积案，深化平安定西、法治定西建设，保持社会和谐稳定。

　　各位代表！今年市政府将继续多渠道筹措资金，为群众办好10件15项实事:1.改造农村危房1.8万户，新建农村户用沼气1650户。2.全面完成市县区本级数字广播电视节目农村覆盖网工程。3.为困难群众办理法律援助案件1200件。4.将100个贫困村列入互助资金试点进行重点扶持。5.改造建设校舍2.3万平方米，改善7所农村薄弱学校办学条件。6.改扩建7所乡镇卫生院，为基层医疗机构培训全科医生70名，为农村1万对计划内怀孕夫妇免费开展孕前优生健康检查。7.建成农村公路通畅工程1100公里，行政村通水泥或沥青路率达到78%以上。8.培训城乡劳动力15万人次。9.解决农村16.26万人安全饮水问题。10.实施老旧供热管网改造34.3公里、供排水管网改造24.3公里，完成背街小巷治理工程8.75公里。

加强法治政府建设

　　各位代表！面对今年繁重艰巨的发展任务，我们将以改革创新精神，牢记宗旨，勤勉尽责，永不懈怠，切实转变政府职能，扎实推进依法行政，努力提高行政效能，全力打造职能科学、权责法定、执法严明、公开公正、廉洁高效、守法诚信的法治政府。

　　一是切实加强学习培训。牢固树立与时俱进的学习理念，倡导各级干部多读书，培养终身学习的意识和自觉性。全面落实市委关于依法治市的实施意见，采取多种形式开展法律培训，切实增强法治信仰，提高各级干部运用法治思维和法治方式深化改革、推动发展、化解矛盾、维护稳定的能力，努力营造良好的法治环境。

　　二是推进政府职能转变。大力简政放权，深入推进行政审批制度改革，切实做好接管放工作，更大激发市场活力，更多释放改革红利。不断健全监管机制，提升政府绩效，切实把政府该管的事管住管好。强化政务服务体系规范化建设，努力营造透明高效、清廉便民的政务服务环境，为企业真正减负，让老百姓办事更加方便，努力把定西打造成行政审批少、服务效率高、发展环境优、市场活力强的投资热土。

　　三是始终坚持依法行政。建立决策科学、执行坚决、监督有力的权利运行体系，深化行政执法体制改革，严格依照法定权限和法定程序行使权力、履行职责，规范行政决策行为。建立市政府部门行政权力清单、市政府部门责任清单、市级财政专项资金管理清单，大力推进电子政务服务网建设，加强政务信息公开。加大责任追究力度，严厉查处执法环节乱作为，严格禁止未经批准乱检查，严肃处理索拿卡要乱收费，着力营造守法诚信的发展环境。自觉接受人大、政协和社会各界监督，促进政府不断提高依法行政效能。

　　四是持续转变工作作风。深入开展“工作落实年”活动，大力推行政府绩效管理，完善重点工作公开承诺、目标管理和考核评价机制，特别是对重点工作的督查，敢于拉下脸面、敢于不留情面、敢于实施问责，快节奏、高质量地推进各项工作落实，切实提高政府各层级的执行力。持之以恒抓好中央八项规定和省、市相关规定落实，巩固扩大教育实践活动成果，加强庸懒散慢拖等问题治理，形成改进作风的常态化机制。从政府领导班子做起，带头密切联系群众，带头改进作风，带头解决实际问题。继续办好电子民生平台，健全有序高效的民意诉求归集办理机制。

　　五是努力做到勤政廉政。严格落实中央和省市有关规定，推进财政预决算公开，严格执行公务接待、公务用车、办公用房标准，严控公务出国(境)，进一步压缩“三公”经费。大力整治机关事业单位“吃空饷”问题，全力推进社会信用体系建设。认真落实党风廉政建设主体责任，支持纪检监察机关严查各类违纪违法案件。坚持以深化改革推进廉政建设，强化廉政风险防控管理。加强行政监察和审计监督，依法治理和纠正损害群众利益的不正之风，抓好公共资源交易、政府投资项目、民生资金等重点领域监管，以勤政廉政实际成效取信于民。

　　各位代表！让我们在党的十八大和十八届三中、四中全会精神指引下，在省委、省政府和市委的坚强领导下，在市人大、市政协的监督支持下，改革创新、求真务实、开拓进取、扎实工作，奋力完成各项目标任务，为加快扶贫攻坚步伐、推动经济社会持续健康发展作出新的更大的贡献！
